# Beyond Spatial Relationships: Residential Greenness and Birth Outcomes

**DOI:** 10.1289/ehp.122-A281

**Published:** 2014-10-01

**Authors:** Wendee Nicole

**Affiliations:** Wendee Nicole was awarded the inaugural Mongabay Prize for Environmental Reporting in 2013. She writes for *Discover*, *Scientific American*, *National Wildlife*, and other magazines.

A growing body of evidence indicates that living near natural, vegetated areas may contribute to various positive health outcomes, ranging from improved mental health to decreased mortality rate.^1^^,^^2^^,^^3^ Past studies have suggested four ways green spaces may improve health: by reducing harmful environmental exposures to noise, heat, or air pollutants; by providing more opportunities for physical activity; by increasing a sense of community belonging and the associated psychosocial benefits; and by directly reducing depression and stress.^1^ In this issue of *EHP* investigators report an association between increased residential greenness and improved birth outcomes, which appeared to be independent of spatially influenced factors including noise and air pollution exposures.^4^

The researchers assessed the relationship between birth weight, preterm birth (categorized as 30–36 weeks or earlier than 30 weeks), and residential greenness among 64,705 singleton births recorded in Vancouver, Canada, from 1999 through 2002. They used the Normalized Difference Vegetation Index (NDVI), a satellite-derived measure, to determine greenness within 100 m of each mother’s home during pregnancy.^4^

**Figure:**
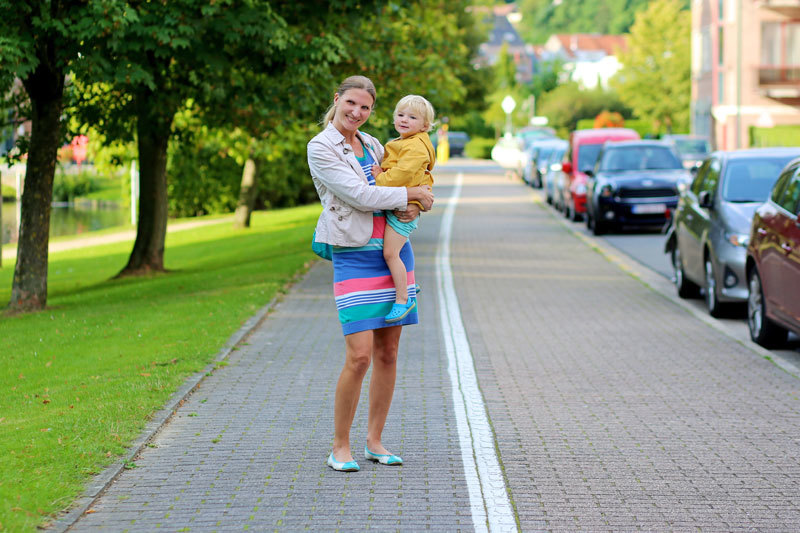
Access to green spaces has been associated with increased physical exercise, but that factor was not associated with improved birth outcomes in the current study. © CroMary/Shutterstock

The authors reported that quartile increases in greenness were associated with higher birth weight for babies born at term (37 weeks or later) and decreased likelihood of having a moderately or very preterm birth, or a small-for-gestational-age baby. Because air and noise pollution can affect birth outcomes, they used detailed models to adjust for these environmental exposures. But in this study living in more “walkable” neighborhoods was associated with lower birth weight and increased risk of preterm birth.^4^

“We thought [noise and air pollution] were the causal pathways of how greenness influenced birth outcome,” says lead author Perry Hystad, an assistant professor of environmental and occupational health at Oregon State University. “We didn’t find that at all … which really suggests other pathways, particularly psychosocial or psychological factors, may be important.” Of the walkability findings, he says, “This really underscores the complexity of how the built environment can influence health and why more research is needed that examines multiple exposures together, rather than each in isolation.”

“The study was well designed and well conducted,” says Payam Dadvand, an assistant research professor at the Centre for Research in Environmental Epidemiology in Barcelona, who was not involved with the study. “Although the study faced limitations in accessing data on some important covariates like indicators of individual socioeconomic status, a wide range of sensitivity analyses conducted by the authors can provide confidence in the findings.”

“This paper did a thorough job of controlling for confounding by the built environment, which strengthens the overall evidence that greenness can positively influence birth outcomes,” says Geoffrey Donovan, a research forester with the Portland Forestry Sciences Laboratory of the U.S. Department of Agriculture Forest Service, who also was not involved with the study.

The present study used an index of greenness ranging from –1 to 1, finding a threshold level of 0.15 above which greenness was associated with improved birth outcomes. No associations were observed under 0.15, which corresponded to dense urban areas, along major roadways, and the downtown core.

“I was intrigued by this finding,” says Donovan. “As far as I know it is the only paper to have done this. My only mild concern is that at the extreme ends of a data set, statistical relationships often have a lot of variability, so it’s best to be very cautious when making inferences. … I’d like to see it replicated before we start talking about policy implications.”

Hystad agrees. “This is where replicating the study in other cities with different NDVI levels—both very low and very high—will be important,” he says.

“Another interesting observation of this study is the suggestion that ethnic minorities might be less likely to benefit from greenness,” says Dadvand. “This is consistent with our study in Bradford, United Kingdom,^5^ where we observed that for white British participants, there was a positive association between residential surrounding greenness and birth weight, whereas for participants of Pakistani origin there was no such association.This requires further investigation by future studies.”

Howard Frumkin, dean of the University of Washington School of Public Health, says greenness is a highly innovative “exposure” in the environmental health sciences. “It’s innovative in that it relates to health promotion, not health threat, and it is a different kind of exposure—not a chemical, but a hard-to-quantify concept of greenness,” he says. “It’s also very actionable; it may be easier to promulgate tree-planting policies in cities than to bring chemical exposures down to very low levels. This is really a game-changing development in the field.”
